# Molecular Detection and Epidemiology of Potentially Zoonotic *Cryptosporidium* spp. and *Giardia duodenalis* in Wild Boar (*Sus scrofa*) from Eastern Spain

**DOI:** 10.3390/ani13152501

**Published:** 2023-08-03

**Authors:** Alba Martí-Marco, Samantha Moratal, Irene Torres-Blas, Jesús Cardells, Victor Lizana, María Auxiliadora Dea-Ayuela

**Affiliations:** 1 Servicio de Análisis, Investigación y Gestión de Animales Silvestres (SAIGAS), Facultad de Veterinaria, Universidad Cardenal Herrera-CEU, CEU Universities, C/Tirant lo Blanc 7, Alfara del Patriarca, 46115 Valencia, Spain; albamartivet@gmail.com (A.M.-M.); samantha.moratalmartinez@uchceu.es (S.M.); jcardells@uchceu.es (J.C.); 2 Wildlife Ecology & Health Group (WE&H), Veterinary Faculty, Universitat Autònoma de Barcelona (UAB), Travessera dels Turons, Bellaterra, 08193 Barcelona, Spain; irene.torres.blas@uab.cat; 3 Departamento de Farmacia, Facultad de Ciencias de la Salud, Universidad Cardenal Herrera-CEU, CEU Universities, C/Ramón y Cajal, Alfara del Patriarca, 46115 Valencia, Spain

**Keywords:** *Cryptosporidium scrofarum*, *Cryptosporidium suis*, epidemiology, *Giardia duodenalis*, molecular characterisation, Spain, *Sus scrofa*, wild boar, zoonoses

## Abstract

**Simple Summary:**

*Cryptosporidium* spp. and *Giardia duodenalis* are widely distributed pathogens in vertebrates. Both protozoa are among the major causes of diarrhoea in humans. Wild boars are known hosts of both parasites and are able to harbour zoonotic species. The main goal of this study was to molecularly evaluate the presence of *Cryptosporidium* spp. and *Giardia duodenalis* in faecal samples taken from hunted wild boar in eastern Spain. This area is experiencing a rapid increase in the wild boar population, which is colonising all habitats, including urban and peri-urban areas, thereby increasing interactions with humans. Both parasites were found in our study, evidencing a high prevalence, mainly of *Cryptosporidium scrofarum* and *Cryptosporidium suis*, which have been previously reported to affect humans. These results point out the potential for wild boar-human transmission because of close contact interactions, such as space sharing or dressing for meat consumption.

**Abstract:**

The protozoans *Giardia duodenalis* and *Cryptosporidium* spp. are common causes of gastrointestinal disease in humans and animals. While both are commonly documented in domestic animals, few studies have analysed their presence in wildlife. To assess the prevalence of both parasites in wild boar (*Sus scrofa*) in the Valencian Community (eastern Spain), 498 wild boar faecal samples were collected from 2018 to 2022. *Cryptosporidium* spp. was detected by performing a nested PCR targeting a 578 bp sequence of the small subunit ribosomal RNA gene (SSU rRNA), followed by sequencing and phylogenetic analysis. For *G. duodenalis*, a qPCR amplifying a fragment of 62 bp from the SSU rRNA was employed. Positive samples were genotyped for glutamate dehydrogenase and β-giardin genes. Different epidemiological factors were considered potential modulating variables in the transmission of both parasites. *G. duodenalis* prevalence was 1.20%, while *Cryptosporidium* spp. prevalence reached 21.7%. Coinfection was observed in 0.2%. Genotyping of *G. duodenalis* isolates only detected genotype E. Two species of *Cryptosporidium* spp. were identified: *Cryptosporidium scrofarum* and *Cryptosporidium suis.* The results of this study demonstrate that the exposure to *Cryptosporidium* spp. in wild boars is high, particularly among young individuals belonging to the Typical Mediterranean climate. Moreover, the probability of infection is dependent on both the season and the density of wild boars. On the other side, exposure to G. duodenalis seems scarce and is influenced, in turn, by the climate. Both *Cryptosporidium* species detected in the present study have been reported in humans. Due to wild boar increasing in number and their colonisation of urban and peri-urban areas, this could represent an inherent health risk for the human population.

## 1. Introduction

European wild boar (*Sus scrofa*) populations have experienced a continuous rise in the last 40 years throughout Europe [1]. The Valencian Community (eastern Spain) is not an exception, with a 159.7% hunting bag increase in the last 10 years, becoming the most popular big game species in the territory [2]. This situation has additional implications, as wild boar are known to carry a plethora of zoonotic pathogens [3,4,5] that can be transmitted to humans when close-contact situations arise, including protozoa. The increase in human–wildlife contacts due to the overlapping of wildlife habitats and human settlements [6,7], handling carcasses while meat dressing [8], and foodborne infections [9,10] are some of the most common scenarios where zoonotic transmission can occur.

*Giardia* and *Cryptosporidium* have been singled out as some of the most important zoonotic protozoa [6]. *G. duodenalis* is one of the most common enteric parasites in humans and domestic animals [7,8]. It is responsible annually for around 280 million cases of human diarrhoea worldwide [9,10], especially affecting children [11,12], and it infects more than 40 animal species [13], particularly in neonates [14]. *Cryptosporidium* spp. is the fifth most important foodborne pathogen, with more than 8 million cases being notified every year [15]. In human medicine, immune-compromised patients can develop a chronic disease, leading to a severe, sometimes fatal, outcome [15,16].

*G. duodenalis* and *Cryptosporidium* have other characteristics that make these parasites some of the most common causes of parasitic diarrhoea in humans [14,15]. The ability of *Cryptosporidium* spp. oocysts and *Giardia duodenalis* to resist conventional water treatments [12,13] and their very low infection dose [16] enhance their risk of transmission [16], as both are mainly foodborne and waterborne parasites. Only ten oocysts of *Cryptosporidium hominis* [17] or *Cryptosporidium parvum* [18,19] or ten cysts of *G. duodenalis* [20] are enough to promote symptomatic disease in healthy human adults. Additionally, there is a lack of effective treatment against *Cryptosporidium* spp. [15,21,22,23], and *Cryptosporidium*’s high environmental resistance is considered a key factor in its transmission [24].

From the eight different assemblages found for *G. duodenalis* (A to H), only A and B have been identified as zoonotic, and both are found in wild boars [25,26]. Among the different species of *Cryptosporidium* described to date, *C. suis* and *C. scrofarum* (formerly known as Crypto pig genotype II [27]) are the most commonly reported from wild boars [28,29,30]. Both species have been detected in human beings, indicating their zoonotic potential [31,32,33,34]. Moreover, swine could act as the origin of contamination of human water and food supplies, as both cryptosporidia species have been found in untreated water [35].

Therefore, given the importance these two protozoa have within the field of Public Health and that both parasites can be found in the ever-increasing population of wild boar, the main aim of this study was to determine the prevalence of both parasites in the wild boar population of the Valencian Community (eastern Spain). Additionally, this study aimed to evaluate the potential effects of climatic, human-related, and host-related factors in their epidemiology.

## 2. Materials and Methods

### 2.1. Study Area and Wild Boar Sampling

The Valencian Community is an autonomous region in eastern Spain. The territory is divided into 31 counties, all of which have a confirmed wild boar presence [2]. Sampling was conducted in areas where hunting activity is allowed (on both private and public land), comprising 1,916,454.75 ha (82.4% of the total territory) [36].

Sampling size (*N*) for each county was calculated based on the expected prevalence of wild boar [37,38,39] over the declared hunting bags [2] using *WinEpi 2.0 Software* [40] (Table 1). Three counties were discarded because the number of wild boars to sample was <1 specimen. Sampling periods involved four game seasons (from October to February) during the years 2018/19 to 2021/22. The total number of collected samples was 498, most of them from hunting events (*N* = 494), while road kills (*N* = 2) and found dead individuals (*N* = 2) were anecdotal. Additional information about the sampled wild boars (location, sex, age, weight, and pregnancy in females) was recorded. Sex was established by observing the genitalia, and age was calculated by dental eruption patterns [41,42].

Refrigerated faecal samples were transported to the Veterinary Faculty CEU-UCH, Alfara del Patriarca (Valencia), where they were kept at 4 °C and processed within 24 h post-collection [37]. After DNA extraction, samples were labelled and stored at −18 °C until further molecular analysis.

### 2.2. Environmental and Population Data Collection

#### 2.2.1. Climate, Rainfall Regime, and Seasonality

According to Köppen–Geiger’s classification [43], the territory can be subdivided into three variants of the Mediterranean climate (Table 1). The Typical Mediterranean (also known as Csa) has moderate winters with rare snowfalls, while summers are dry and hot, with temperatures above 30 °C. The Continental Mediterranean climate (Csa-Bsk) has frequent snowfalls during the winter but experiences long, dry summers, even reaching 40 °C. The Dry Mediterranean (Bsh-Bsk) is located in the southernmost area of the territory, where winters are mild (around 10 °C) and the maximum temperature in summer is above 30 °C.

The rainfall regime is concentrated in spring and autumn, usually in a few days with heavy rains [44,45], while drought is common during the summer. The maximum annual rainfall is 800 mm, commonly seen in some coastal mountain ranges and in the northwestern extremity of the territory. On the southern end, where the climate is dry, the annual rainfall value is around 300 mm, which is the minimum annual rainfall value of the Valencian Community [45]. To analyse the effect of the climate on parasite presence, the sampled counties were clustered according to the bioclimatic area they mainly belong to and the average rainfall of the sampled municipalities [46].

Sampling dates were registered in order to classify the samples according to the astronomical-meteorological seasons in the Northern Hemisphere (spring includes samples taken from mid-March to mid-June, summer from mid-June to mid-September, autumn from mid-September to mid-December and winter from mid-December to mid-March) [47].

#### 2.2.2. Land Use

Land use allows us to classify the territory into urbanised, irrigated cropland (mainly citrus production and growing vegetables), rain-fed crops (olive, carob, and almond trees), and forest land (almost 60% of the total) [36]. The origin of the animals was registered at the municipal level to know the possible effect of land use on parasite prevalence.

Crop fertilisation is a common way to dispose of the slurry from pig farms, which can carry *Giardia* sp. and *Cryptosporidium* sp. infective cysts. But transport costs limit the use to a close buffer from the origin [48]. Counties were classified according to the presence or absence of this practice.

#### 2.2.3. Wild Boar Population

The geographic information about the origin of the samples allowed us to compare the effect of wild boar population density in the above-mentioned parasites. The territory was classified into three categories based on hunting bags: sustainable density with 0.3–1 wild boar/km^2^; high density from 1.1 to 3 wild boar/km^2^; and extreme density, 3.1–6.7 wild boar/km^2^, in agreement with the classification made by local authorities [2]. Age groups were established in piglets (≤6 months old (m.o.), striped coat), juveniles (7 to 12 m.o., reddish colour), sub-adults (12 to 18 m.o.), and adults (≥18 m.o.) by dental eruption and coat colour patterns [49,50].

#### 2.2.4. Human Population

To evaluate the potential relationship between wild boar positivity and a possible anthropogenic disturbance, the territory was classified according to human density. Counties were categorised as rural (<100 inhabitants/km^2^), intermediate (100–499 inh/km^2^), and urban (>500 inh/km^2^) [51] (Table 1).

### 2.3. DNA Extraction

DNA extraction was performed within 24 h after sampling using the QIAamp^®^ DNA Stool Mini Kit (QIAGEN, Hilden, Germany) according to the manufacturer’s instructions.

### 2.4. Molecular Detection and Characterisation of Giardia duodenalis and Cryptosporidium spp.

Samples were tested for *G. duodenalis* presence with a real-time PCR (qPCR) protocol, amplifying a 62 bp segment of the small subunit ribosomal RNA (SSU rRNA) gene of the parasite [52]. Briefly, 3 µL of template DNA were used in a total volume reaction of 25 µL [12.5 pmol of each primer (Gd-80F/Gd-1278R), 10 pmol of the probe, and 12.5 µL of NZY Supreme qPCR Probe Master Mix (Nzytech genes and enzymes, Lisbon, Portugal)]. The detection of parasitic DNA was performed on an AriaMx real-time PCR (qPCR) instrument (Agilent Technologies, Santa Clara, CA, USA) following the already described amplification protocol [52,53]. A negative control without a template DNA and a positive control (*G. duodenalis* genotype C isolated from an infected dog) were used in each reaction.

After qPCR, positive samples were further subjected to semi-nested PCRs for glutamate dehydrogenase (*gdh*) [54] and β-giardin (*bg*) [55] specific parasite genes.

The presence of *Cryptosporidium* spp. was assessed by means of a nested PCR amplifying a 578 bp fragment from the SSU rRNA gene [56]. A total of 3 µL of DNA samples were used in a 25 µL amplification reaction, containing 12.5 pmol of each primer pair (18SicF2/18SicR2 and 18SicF1/18SicR1). Both PCR amplification reactions were carried out in a thermal cycler GeneAmp PCR System 2700 (Applied Biosystems, Foster City, CA, USA) [54]. All the PCR conducted included negative and positive controls, the latter being DNA from *Cryptosporidium ubiquitum*-positive farmed lamb. Products of positive samples with a band of the expected size were visualised on 1.5% agarose gel pre-stained with RedSafe TM Nucleic Acid Staining Solution (iNtRON Biotechnology, Seongnam, Republic of Korea).

### 2.5. Sequencing and Phylogenetic Analysis

Positive samples were sequenced by an external sequencing service (Genomics Department at Principe Felipe Research Centre, Valencia, Spain). The quality control and assembly of chromatograms were conducted using Chromas version 2.6.6 (Technelsyum DNA Sequencing Software, South Brisbane, QLD, Australia). The resulting sequences were blasted against *Cryptosporidium* spp. and *G. duodenalis* sequences available in the NCBI GenBank database using the online BLAST tool (http://blast.ncbi.nlm.nih.gov/blast, accessed on 15 January 2023). A phylogenetic analysis was applied to the *Cryptosporidium* spp. SSU rRNA partial sequences were obtained, which exhibited <100% identity with the closest reference sequence, using the MEGA X 10.1 [57]. The partial sequences were aligned with selected *Cryptosporidium* species sequences retrieved from Genbank, distance matrices were calculated, and the phylogenetic tree was inferred by the Maximum Likelihood (ML) method (bootstrap test on 1000 replicates). *Cryptosporidium* spp. partial sequences obtained were deposited in GenBank under the accession numbers OR030357–OR030362 and OR030363–OR030373.

### 2.6. Data Analysis

Statistical analysis was performed using the R programme and RStudio Version 4.1.0 [58]. From the original database containing 498 individuals, 24 wild boars were excluded because of incomplete data. Therefore, the final analysis was carried out using a total of 474 individuals with complete datasets. To assess the correlation between explanatory variables, different types of statistical tests were used depending on the nature of the data. Briefly, Cramer’s V index was used when both explanatory variables were categorical; Pearson’s product–moment correlation (Pearson’s correlation coefficient) was applied when both explanatory variables were numerical; and the Kruskall–Wallis rank sum test was used to assess the correlation between numerical and categorical variables.

Data regarding the positive status of *Giardia duodenalis* or *Cryptosporidium* spp. were analysed separately and by applying different statistical models due to the difference in the number of positive animals for *Giardia duodenalis* (5/474) and *Cryptosporidium* spp. (109/474).

#### 2.6.1. *Giardia duodenalis*

Binary logistic regression was used to examine differences in the presence or absence of *Giardia duodenalis*. A backward stepwise model selection process was applied to select the most parsimonious model, using Akaike’s criteria (AIC) as the election factor. The variables included in the saturated models were sex, the interaction between age category and wild boar density, the type of human population, climate, slurry, and the sampling season. The backward stepwise selection was performed using the “step AIC” function that can be found within the “MASS” R 7.3-60 package [59]. McFadden R2 was calculated using the “pscl” package from R [60] for each final chosen model to assess model fitting. The importance of each predictor variable in the final model was assessed using the varImp function from the “caret” package [61]. Finally, the Variance Inflation Factor (VIF) was calculated for each final model to test for the presence of multi-collinearity, also using the “caret” package [61].

#### 2.6.2. *Cryptosporidium* spp.

A Classification and Regression Tree (CART) model was applied to determine the contribution of the following explanatory variables in testing positive for *Cryptosporidium* sp.: sex, age category, wild boar density, climate, use of nitrogen as fertiliser, and the sampling season. As the variable response is binomial (positive/negative), a classification tree was built to fit the data. Two of the main problems faced by this type of model are finding good data splits and data overfitting [62]. In our analysis, the information gain criteria were applied to determine the best split, and the complexity parameter was used to prune the tree and thus avoid data overfitting. Assessment of model reliability was performed by calculating the prediction error rate (accuracy test). The “rpart” library [63] was used to fit the classification tree, and its graphical representation was conducted using the “rpart.plot” library [64].

## 3. Results

The total number of wild boars sampled was 498. The prevalence found for *G*. *duodenalis* and *Cryptosporidium* spp. was diverse depending on the different study variables, which were considered possible modulators for the transmission of both parasites (Table 2).

### 3.1. Giardia duodenalis

The general prevalence was 1.2% (6/498; CI 95% 0.3–2.2%). The median values of the generated cycle threshold (Ct) were 34.7 (range: 25.9−39.8). Genotyping was successful in only one sample by using the gdh gene, resulting in the assemblage E, typical of the artiodactyl order [65].

After assessing the correlation between explanatory variables, those selected for the model were climate (in order to consider vegetation cover and environmental humidity) as a factor directly related to the cyst’s survival [66]; fertilisation with slurry, an indicative value of potential cross-contamination from pig farms [67]; and, finally, the season in relation to temporary environmental conditions.

The simplest model able to explain the maximum variability of the data, according to backward selection and AIC, included only the variable climate (Table 3).

**Figure 1:**
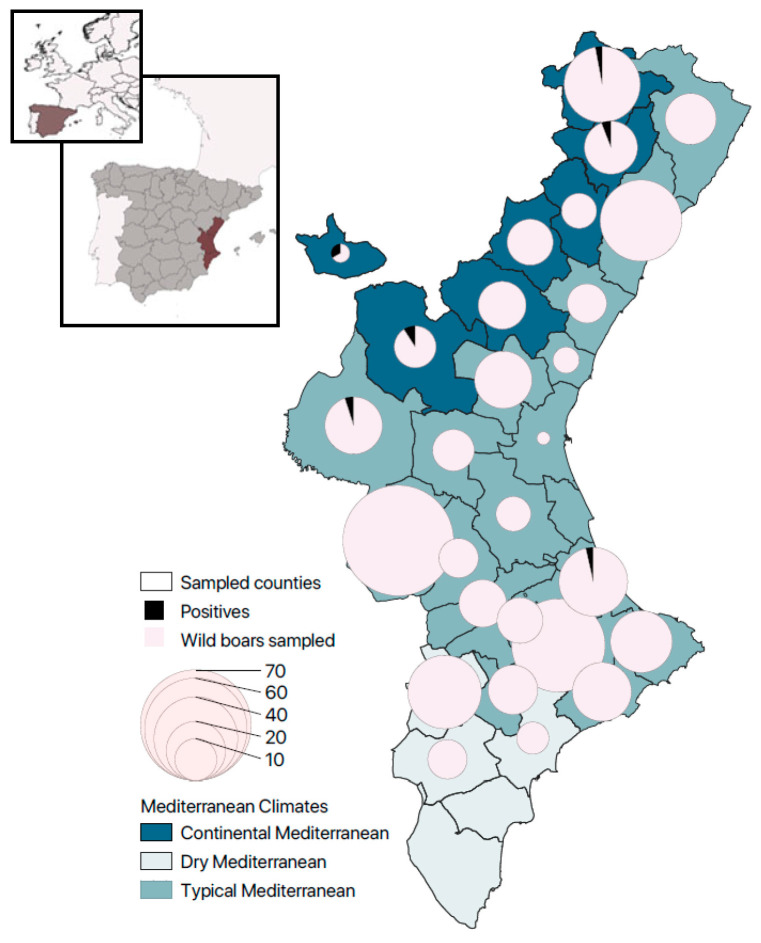
Insert: Location of the Valencian Community and Spain in relation to the European continent. Main map: Relationship of positives to *G. duodenalis* over the total sampled animals per county, classified according to the climate. Number of positive animals is shown in the map as the total number of wild boars (*n*) that were positive for *G. duodenalis*/total number of wild boars sampled in the county.

Observed prevalences for the Continental Mediterranean, Typical Mediterranean, and Dry Mediterranean were 4.3% (4/93; CI 95% 0.2–8.4%), 0.6% (2/359; CI 95% 0.0–1.3%), and 0% (0/46; CI 95% 0.0–6.3%), respectively. There are significant differences (*p*-value = 0.015) between Continental and Typical Mediterranean climates; no other significant differences among climates were detected (Table 3 and Figure 1).

### 3.2. Cryptosporidium spp.

**Figure 2:**
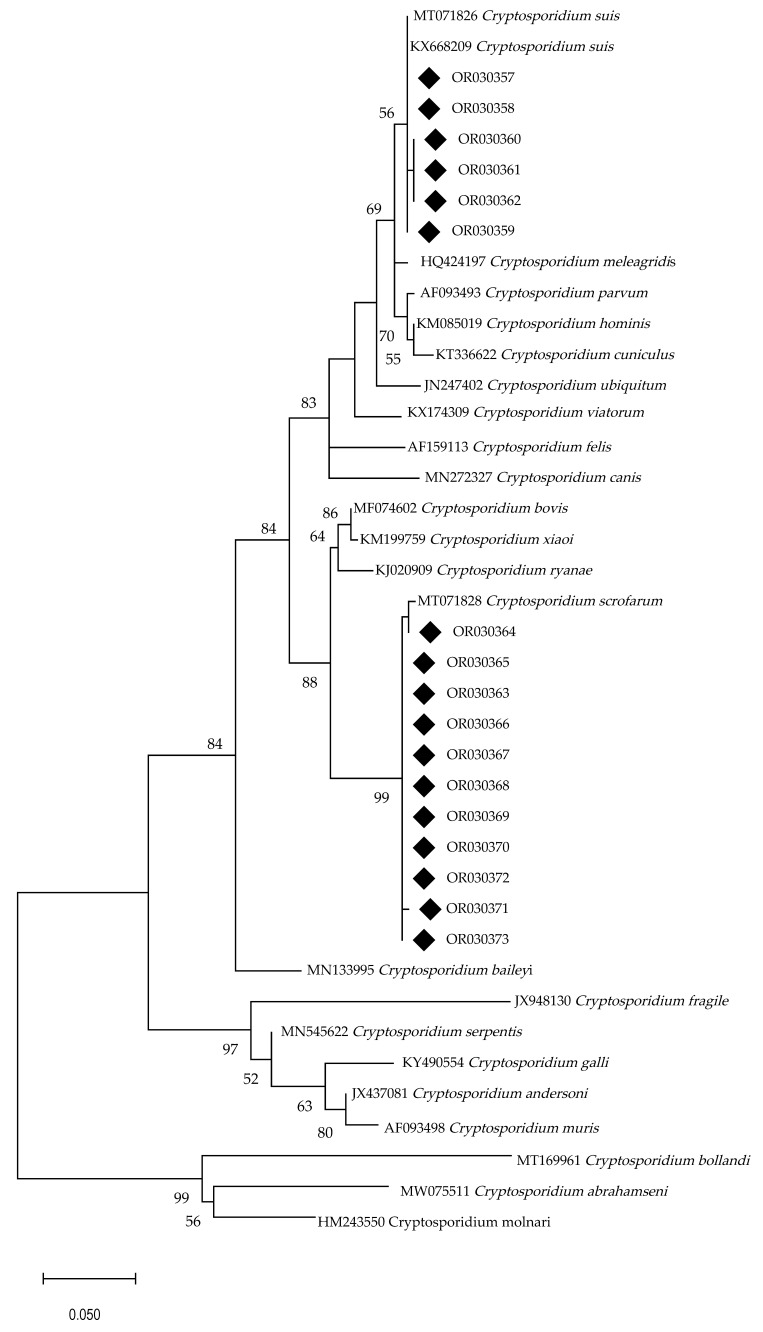
Phylogenetic relationships for *Cryptosporidium* SSU rRNA partial sequences from the present study (◆). The model was inferred by the Maximum Likelihood (ML) method based on the T92+G substitution model. The tree with the highest likelihood (−1859.60) is shown. The percentage support (>50%) for each cluster is indicated at the left of the supported node. The tree is at scale, with the scale bars referring to the phylogenetic distance expressed in nucleotide substitutions per site.

The general prevalence of *Cryptosporidium* was 21.7% (108/498; CI 95% 18.1–25.3%). BLAST results revealed two *Cryptosporidium* species present, namely *C. suis* (2.8%; 14/498; CI 95% 1.4–4.3%) and *C. scrofarum* (18.9%; 94/498; CI 95% 15.5–22.3%) (Table 4). The phylogenetic reconstruction supports BLAST results. The 11 identified *C. scrofarum* isolates clustered with the reference sequence MT071828, while the six *C. suis* isolates formed another cluster with the reference sequences MT071826 and KX668209. Despite the relatively small size of the sequenced regions, the overall topology of the tree is consistent with the known topology for the *Cryptosporidium* genus (Figure 2).

Coinfections between both species could not be assessed with the deployed methods. One mixed infection among *G. duodenalis* and *C. scrofarum* was detected (0.2%, CI 95% 0.00–0.591%).

**Figure 3:**
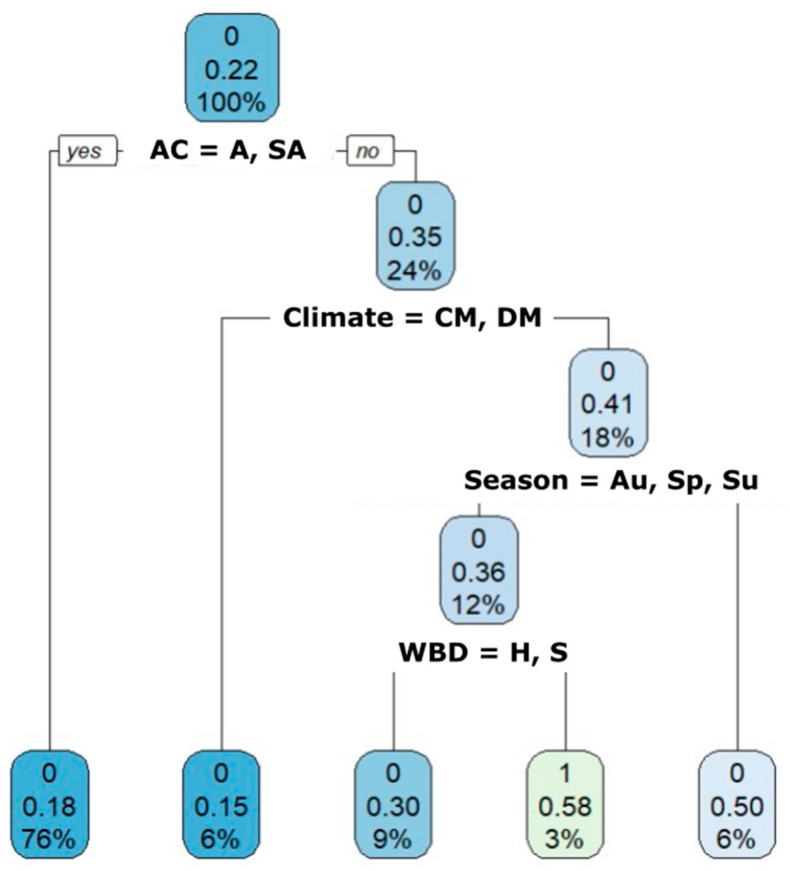
Tree-based classification modelling evaluates the relevance of different risk factors related to *Cryptosporidium* spp. infection. Abbreviation key: **AC**: Age Category. A: Adult (>24 m); SA (12–24 m): Sub-adult; *p* (0–6 m): Piglet; J (6–12 m): Juvenile. **Climates**: Continental Mediterranean (CM), Dry Mediterranean (DM), Typical Mediterranean (TM). **Seasons**: Au: Autumn; Sp: Spring; Su: Summer; Wi: Winter. **WBD**: Wild Boar Density. H: High; S: sustainable; E: extreme.

**Figure 4:**
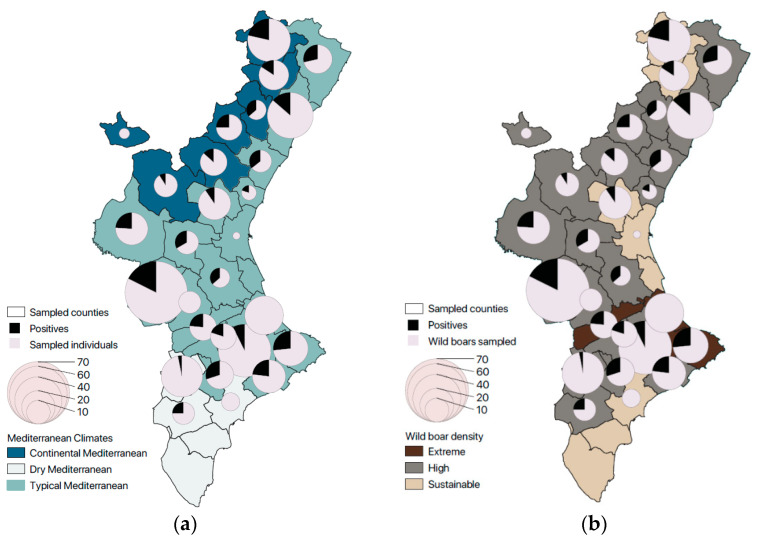
Relation of positives to *Cryptosporidium* spp. over the total sampled animals per county, (**a**) classified according to the climate and (**b**) according to wild boar density.

The most parsimonious tree model to predict the probability of testing positive for *Cryptosporidium* spp. (*p* = 0.22) was fitted using four variables (i.e., age, climate, season, and wild boar density) (Figure 3). Independently of climatic conditions, seasonality, and wild boar density, piglets and juveniles have higher probabilities (*p* = 0.35) of testing positive than adults and sub-adults (*p* = 0.18). Within piglets and juveniles, those living in areas with a Typical Mediterranean climate (TM) have a higher probability of being positive (*p* = 0.41) than those living in Continental and Dry Mediterranean counties (*p* = 0.15). In turn, in areas with TM, the infection probability for piglets and sub-adults depends on the season, being higher in winter (*p* = 0.50). During the other seasons, infection probability is dependent on wild boar density (i.e., higher probability in counties with extreme density) (Figure 4).

## 4. Discussion

To the authors’ knowledge, this is the first time that the presence of *Giardia duodenalis* and *Cryptosporidium* spp. in wild boar has been comprehensively studied in the Valencian Community (eastern Spain). We detected a high prevalence of *Cryptosporidium* spp. and a low prevalence of *G. duodenalis*. Moreover, the role of different epidemiological variables has been assessed, identifying potential risk factors in *Cryptosporidium* spp. transmission.

There are great differences in the prevalence of *Giardia duodenalis* (1.2%; 4/498) and *Cryptosporidium* spp. (21.7%; 108/498). Both parasites have simple life cycles and low infective doses (only 10 (oo)cysts [19,20]). Thus, it is crucial to study host- or environmental-related factors that can explain the differences in the observed prevalence. Among them, climate seems to be the most plausible, comparing the relative resilience of *Cryptosporidium* spp. oocysts and the vulnerability of *Giardia duodenalis* cysts to some environmental stressors, essentially dryness and UV radiation exposure [66,68,69].

The overall observed prevalence for *G. duodenalis* is similar to other results reached by previous studies conducted in the Iberian Peninsula (1.3% in Galicia or 0% in Portugal) [37,70]. A slightly higher prevalence (i.e., 5.6%) has been detected in a recent study that includes samples from the different bioregions of Spain, probably because this study encompasses regions with high climatic diversity [71]. The highest prevalence in the Iberian Peninsula was detected in Cordoba (southern Spain), where the positives reached 22.5% [39]. In the European context, previous studies have found a similar prevalence (1–4%) to our results [72,73]. Generally, the differences in *G. duodenalis* prevalence between studies could mainly be related to climatic conditions as well as sampling data (due to variations in the temperature and rainfall regime). In accordance with this argument, in our dataset, the climate is the most relevant variable related to *Giardia* prevalence (Table 3), with significant differences observed between Continental Mediterranean and Typical Mediterranean climates. Higher prevalence was found in counties with CM climates, probably related to the pluviometric regime (as high as 800 mm) that enables the growth of denser vegetation cover. Under these conditions, *G. duodenalis* cysts, which are highly susceptible to drying, extreme temperatures, and UV radiation [73], are more likely to survive. It is known that *G. duodenalis* cysts remain infective for several months in humid and fresh areas, enabling rapid accumulation in the environment [74]. Another key factor in *G. duodenalis* infectiveness, closely influenced by the pluviometric regime, is the presence of water points such as ponds and dams. In these water sources, cysts survive up to 56 days from 0 °C to 7 °C or up to 28 days at 17–20 °C. The survival period is even more prolonged in rivers (84 days at 0–4 °C and 28 days between 20 and 28 °C) [74]. It is worth noting that rivers can constitute a potential point of pathogen transmission, as both wildlife and humans make use of them.

However, due to the very low prevalence of *G. duodenalis* found in the sampled populations of wild boar, statistical results must be interpreted with caution.

Only one of the positive samples (1/6) was successfully genotyped with glutamate dehydrogenase (*gdh*) and was found to be assemblage E. None of the positive samples could be genotyped with beta-giardin (*bg*). Similar amplification rates have been observed in wild mammals in previous studies [39,71,75,76,77], in contrast to other groups, like humans or birds [78,79], with higher rates of success. Artiodactyls are a host type for assemblage E [65]. However, this assemblage has been occasionally detected in humans from Europe [80] and developing countries [81]. Therefore, some authors consider it zoonotic [80].

The general prevalence of *Cryptosporidium* spp. (21.7%) is higher in comparison to previous studies carried out in northwestern and southern Spain (6–8%) or in Portugal (1.4%) [30,37,39,70,75]. Local climatic conditions in eastern Spain (mild temperatures and the regulating effect of the Mediterranean Sea) lengthen the survival of the oocysts [74], thus enhancing the risk of transmission. Wild boar populations have been surging in numbers and expanding their range during the last few years. Population growth favours disease transmission among suids, and close interactions with human beings (hunting, butchering, and dressing [82]) enhance the risk of disease transmission.

Age was the most relevant factor related to prevalence. Piglets and juveniles were the most susceptible groups to infection; this result is consistent with previous studies showing decreasing *Cryptosporidium* spp. prevalences with age. Furthermore, susceptibility to different *Cryptosporidium* species appears to differ between groups based on age [83,84,85]. Additionally, *Cryptosporidium* spp. infections seem to be modulated by climate and season. The Mild Typical Mediterranean climate shows the highest prevalence, probably related to favourable environmental conditions for oocyst survival, in comparison to the more challenging Continental and Dry Mediterranean climates [86]. Winter is the season with the highest probability of *Cryptosporidium* spp. infection, presumably related to changes in the behaviour that favour transmission (aggregation, group mixing, increased travelling, and physical contact) due to mating season [87].

In coincidence with previous studies, wild boar density was also an element involved in the observed results [88]. In our study area, extreme densities (established at >3.1 wild boars/km^2^ and even reaching 6.7 wild boars/km^2^ in some counties) [2] increase the prevalence of the parasite over the expected prevalence (enhancing contact among individuals). This constitutes a risk factor for environmental and public health.

The two detected species, namely *C. suis* and *C. scrofarum*, are the main *Cryptosporidium* species found in wild boars, with similar proportions registered in Central European countries like Austria, the Czech Republic, Poland, and Slovakia [28,29]. Both have been reported in human beings [31,32,33,34]; therefore, wild boars could potentially act as an important source of infection for people. *C. scrofarum* is the predominant one in this species, as shown by prior studies [29,30,39,71], infecting all wild boar age groups [30].

Previous studies have found (oo)cysts of *Cryptosporidium* spp. and *G. duodenalis* in leachate from croplands related to the farm industry [89,90,91,92]; hence, we considered the fertilisation with slurry from pig farms and main land use from the sampled areas as potential risk factors worth investigating. Although our results showed these variables were not statistically significant, this must be cautiously interpreted. In this study, information concerning the presence/absence of pig farms surrounding the sampling areas was not accessible. Therefore, we relied on data indicating the use of slurry as a natural fertiliser, although quantities, previous treatments, and its origin were not available. Further investigation is needed to discern the role that malpractices related to manure management may play in determining the transmission risk of *G. duodenalis* and *Cryptosporidium* spp.

## 5. Conclusions

The results of this study emphasised the importance of expanding *Cryptosporidium* spp. and *G. duodenalis* monitoring beyond domestic species, including wildlife, in disease surveillance programs. Further studies might be necessary to evaluate the potential effect of fertilisation with slurry on the microbiological contamination of croplands and its transference to humans, domestic animals, and wild animals. It is necessary to further investigate the connections between pathogens, environmental factors, human activities, and wildlife as a way to prevent future outbreaks from a health perspective.

## Figures and Tables

**Table 1:** Description of the sampling area and achieved samples. Wild boar population determined the minimum sampling size. Dimensions, climatic characteristics, and human influence (human density, use of the land, and fertilisation with slurry) in the given counties are shown.

County	Climate	Human Density	Land Use	Slurry	Hunting Area (ha)	Wild Boar Hunting Bag	Wild Boar Density	Minimum Sample Size	Achieved Sample Size
Alto Mijares	CM	R	F	1	53,261.19	857	H	6.7	12
BaixMaestrat	TM	R	F-I	1	104,635.04	1480	H	11.6	15
Els Ports	CM	R	F	1	79,599.80	731	S	5.8	33
L’Alcalatén	CM	R	F-I	0	62,539.34	712	H	5.6	7
Alt Maestrat	CM	R	F	1	64,532.02	518	S	4.1	16
Plana Alta	TM	I	F-I	1	74,611.73	1283	H	10.1	38
Plana Baixa	TM	I	I	0	47,337.87	544	H	4.3	9
Alto Palancia	CM	R	F-I	0	87,353.56	1292	H	10.2	13
**TOTAL CASTELLÓN**					**573,870.59**	**7416**		**58.4**	**143**
Hoya de Buñol	TM	R	I	0	74,865.52	913	H	7.2	10
Ribera Alta	TM	I	I	1	80,757.26	1021	H	8.0	7
Camp de Morvedre	TM	I	I	0	19,424.64	317	H	2.5	4
Camp de Turia	TM	I	F-I	1	64,412.67	518	S	4.1	19
Rincón de Ademuz	CM	R	F	0	36,663.14	490	H	3.9	2
Valle de Cofrentes-Ayora	TM	R	F	1	123,981.80	2765	H	21.8	70
L’Horta	TM	U	I	0	15,956.24	95	S	0.7	1
Canal de Navarrés	TM	R	F	0	55,325.44	577	H	4.5	9
La Costera	TM	I	F-R	0	45,618.45	1216	E	9.6	13
La Plana de Utiel-Requena	TM	R	R	1	162,835.77	2799	H	22	19
Ribera Baixa	TM	I	I	-	14,854.79	87	S	0.7	-
La Safor	TM	I	I	0	30,855.04	2399	E	11	27
La Valld’Albaida	TM	I	F-R	1	68,677.48	916	H	7.2	12
Los Serranos	CM	R	F	1	144,613.83	1393	H	11	10
**TOTAL VALENCIA**					**938,842.14**	**14,505**		**114.1**	**203**
Baix Segura	DM	I	R	-	40,695.07	149	S	1.2	-
BaixVinalopó	DM	U	R	-	18,503.75	62	S	0.5	-
El Comtat	TM	R	F	0	35,521.49	878	E	6.9	50
VinalopóMitjà	DM	I	R	0	58,736.34	894	H	7	9
L’Alacantí	DM	U	R	0	39,683.17	349	S	2.7	6
L’Alcoià	TM	I	F-R	1	46,424.67	1099	H	8.6	14
L’AltVinalopó	DM	R	R	1	56,473.58	739	H	5.8	31
Marina Alta	TM	I	F-I	0	56,811.46	2034	E	16	22
Marina Baixa	TM	I	F	0	43,580.76	975	H	7.7	20
**TOTAL ALICANTE**					**396,430.34**	**7179**		**56.5**	**152**
**TOTAL VC**					**1,909,143.08**	**29,100**		**229**	**498**

Abbreviation key: **Climate:** CM: Continental Mediterranean; DM: Dry Mediterranean; TM: Typical Mediterranean. **Human Density:** R: Rural; I: Intermediate; U: Urban. **Land use:** F: Forested; I: Irrigated; R: Rainfed. **Slurry**: 1: yes; 0: no. **Wild boar density:** S: Sustainable; H: High; E: Extreme. **VC**: The Valencian Community.

**Table 2:** Prevalence of infection by *Giardia duodenalis*, *Cryptosporidium suis,* and *Cryptosporidium scrofarum* in wild boars (*Sus scrofa*) in relation to the studied variables.

		*Giardia duodenalis*	*Cryptosporidium suis*	*Cryptosporidium scrofarum*
**Climate**	CM	4.3% (4/93)	1.1% (1/93)	23.7% (22/93)
	TM	0.6% (2/359)	3.3% (12/359)	19.5% (70/359)
	DM	0% (0/46)	0% (0/46)	8.7% (4/46)
**Season**	Summer	3.22% (1/31)	0% (0/31)	6.5% (2/31)
	Autumn	0.5% (1/209)	1% (2/209)	22.9% (48/209)
	Winter	0.5% (1/195)	3.6% (7/195)	18.5% (36/195)
	Spring	0% (0/63)	4.8% (3/63)	12.7% (8/63)
**Land use**	Forested land	1.3% (4/307)	2.3% (7/307)	20.1% (64/307)
	Irrigated land	0.9% (1/108)	4.6% (5/108)	18.5% (20/108)
	Rainfed land	1.2% (1/83)	1.2% (1/83)	14.5% (12/83)
**Human** **density**	Rural	1.7% (5/297)	8.4% (7/83)	65.1% (54/83)
	Intermediate	0.5% (1/194)	3.1% (6/194)	21.6% (42/194)
	Urban	0% (0/7)	0% (0/7)	0% (0/7)
**Wild boar** **density**	Sustainable	2.7% (2/75)	0% (0/75)	18.7% (14/75)
	High	0.9% (3/311)	3.9% (12/311)	21.5% (67/311)
	Extreme	0.9% (1/112)	0.9% (1/112)	13.4% (15/112)
**Age group**	Piglet	0% (0/30)	6.7% (2/30)	30% (9/30)
	Juvenile	0% (0/83)	2.4% (2/83)	31.3% (26/83)
	Sub-adult	2.7% (1/37)	0% (0/37)	13.5% (5/37)
	Adult	1.2% (4/324)	3.1% (10/324)	15.1% (49/324)
	Unknown	4.2% (1/24)	0% (0/24)	20.8% (5/24)
**Sex**	Male	1.3% (3/244)	3.6% (8/244)	19.6% (44/244)
	Female	0.8% (2/237)	2.5% (6/237)	19.8% (47/237)
	Unknown	5.9% (1/17)	0% (0/17)	17.6% (3/17)

**Table 3:** Model results for *G. duodenalis*. Reference category: Continental Mediterranean.

Factor	Estimate	S.E.	*p*-Value
Dry Mediterranean	−17.48	2672.95	0.99
Typical Mediterranean	−2.74	1.12	0.015

**Table 4:** *Cryptosporidium* spp. isolates are classified per species according to the closest reference sequence.

Species	N	Reference Sequence	% ID	SNV	Accession Number
***C. suis***	6	MT071826	100	None	OR030357– OR030362
	3	MT071826	99.52–99.62	InsT; T → A	
	3	KX668209	99.78–99.81	DelA	
	1	MT561508	100	None	
***C. scrofarum***	82	MT071828	100	None	OR030363– OR030373
	5	MT071828	99.78–99.8	T → Y	
	4	MT071828	99.8–99.81	T → C	
	1	MT071828	99.8	A → R	
	1	MT071828	99.56	C → T; T → C	

ID: identity; SNV: single nucleotide variant; Ins: insertion; Del: deletion.

## Data Availability

Not applicable.
